# Evaluation of Costs, Efficiency and Performance of a Pilot Genetic Testing Traceback Program for Ovarian Cancer

**DOI:** 10.3390/cancers18142292

**Published:** 2026-07-16

**Authors:** Dina Hassen, Adam H. Buchanan, Nora B. Henrikson, M. Cabell Jonas, Kathleen A. Leppig, Tracey Leitzel, Aaron Scrol, Adrienne N. Deneal, Daniela Canedo, Anastatia M. Kunnmann, Alanna Kulchak Rahm, Jing Hao

**Affiliations:** 1 Geisinger, 100 N. Academy Ave., Danville, PA 17822, USAjhao@geisinger.edu (J.H.); 2 Kaiser Permanente Washington Health Research Institute, 1730 Minor Ave., Suite 1360, Seattle, WA 98101, USA; 3 Mid-Atlantic Permanente Research Institute, 700-B 2nd Street NE, Washington, DC 20002, USA; 4 Division of Medical Genetics, Department of Medicine, University of Washington, 1705 NE Pacific St., Seattle, WA 98195, USA; 5 National Institutes of Health, 9000 Rockville Pike, Bethesda, MD 20892, USA

**Keywords:** ovarian cancer, implementation cost, economic evaluation, oncology organizations

## Abstract

Background/Objectives: Guideline-issuing organizations recommend that women with ovarian cancer undergo genetic testing to identify genetic variants related to hereditary breast and ovarian cancer. The National Cancer Institute proposed the Traceback framework to conduct genetic testing on previously untested women and their at-risk relatives. We implemented a pilot Traceback program in three large healthcare systems: Geisinger (GE), Kaiser Permanente Washington (KPWA), and Kaiser Permanente Mid-Atlantic States (KPMAS). We evaluated the performance, cost, and efficiency of the implementation. Methods: We developed three decision-analytic models representing implemented programs to evaluate performance and estimate direct implementation costs and efficiency from a healthcare system perspective using a bottom-up micro-costing approach based on real-world data. We generalize our findings by modeling hypothetical Traceback programs based on our implementation using national cost data and literature estimates. Results: The programs identified 450, 240, and 878 living ovarian cancer patients at GE, KPWA, and KPMAS, respectively. Of those, 224 (49.8%), 149 (62.1%), and 224 (25.5%) were previously untested, of which 18 (8.0%), 37 (24.8%), and 74 (33.0%) underwent genetic testing. The program costs were $41,564 (GE), $97,396 (KPWA), and $120,327 (KPMAS), with KPMAS showing the lowest cost per patient tested and cost per case detected ($1626, $17,190) versus ($2309, $41,564) (GE) and ($2632, $48,698) (KPWA). Conclusions: Traceback programs were feasible to implement and were associated with improved genetic testing uptake for ovarian cancer patients in this non-randomized pilot. Evaluable outcomes reflect proband testing; additional data and evaluations are needed for assessing cascade testing. Quantifying costs can inform decision-makers on resource allocation for similar programs.

## Simple Summary

Ovarian cancer is associated with poor health outcomes that are often a result of late diagnosis. Since a portion of ovarian cancer cases can be explained by hereditary conditions, genetic testing of patients with ovarian cancer and their at-risk relatives is recommended to identify those who may benefit from preventive measures and surveillance for early detection. Traceback programs aim to identify and test patients with ovarian cancer and their at-risk relatives who have not undergone standard genetic testing. We estimated and compared the performance, cost and efficiency of a pilot Traceback program implemented at three integrated health systems. Program cost ranged from $41,564 to $120,327, with the cost per patient tested ranging from $1626 to $2632 and the cost per case detected ranging from $17,190 to $48,698. Although additional data and evaluations are needed to fully assess cascade testing, we found Traceback program implementation to be efficient and successful in genetic testing of ovarian cancer patients.

## 1. Introduction

Ovarian cancer is the third most common and most fatal gynecological cancer [1,2]. In the United States, it is the fifth leading cause of cancer-related deaths; an estimated 20,890 new cases are expected in 2025 [3]. Overall, less than half of women diagnosed with ovarian cancer survive five years following diagnosis, with late detection contributing to the poor prognosis [4]. Over 75% of cases are diagnosed at more advanced stages due to asymptomatic early-stage disease and nonspecific symptoms of late-stage disease [4].

The strongest risk factor for ovarian cancer is family history, with familial genetic syndromes contributing to 10% to 12% of ovarian cancers [4,5,6]. The majority are explained by pathogenic/likely pathogenic (P/LP) germline *BRCA1/2* gene variants, which account for almost 40% of ovarian cancer cases in women with a family history of the disease [7,8]. The estimated lifetime risk of breast cancer in women with a P/LP *BRCA1/2* variant is over 80%; lifetime ovarian cancer risk is estimated to be 54% for those with a P/LP *BRCA1* variant and 23% for those with a P/LP *BRCA2* variant [9]. Since *BRCA1* and *BRCA2* variants are inherited in an autosomal dominant manner, first-degree relatives of those with a variant have a 50% chance of sharing the familial disease-causing variant.

These factors underscore the importance of genetic testing for *BRCA1/2* and other hereditary cancer genes in women with a personal history of ovarian cancer as well as cascade testing in their at-risk relatives [10,11,12,13]. Despite recommendations by The National Comprehensive Cancer Network (NCCN) and other guideline-issuing organizations including the American College of Obstetrics and Gynecologists (ACOG) and the Society of Gynecologic Oncology (SGO) for genetic testing of women with ovarian cancer at any age, uptake remains low due to systemic barriers at the organizational level [10,12,14]. To increase access to genetic testing, the National Cancer Institute proposed the Traceback framework for genetic testing of women with ovarian cancer and their at-risk relatives.

While there have been studies to inform the cost-effectiveness [15,16] of genetic and cascade testing on women with ovarian cancer, implementation costs are an important but underreported driver for organizational decision makers when determining whether an evidence-based practice (EBP) will be adopted [17]. Despite growing calls to include costs as an outcome in implementation science research, multiple reviews have found that cost outcomes are rarely evaluated [18,19,20].

We implemented a multisite, non-randomized pilot Traceback program in three large healthcare systems to (1) identify women with ovarian cancer who had not undergone current standard genetic testing, (2) offer and conduct genetic testing on identified probands, and (3) facilitate cascade testing among at-risk proband relatives under the Feasibility and Acceptability of Cascade Traceback Screening (FACTS) study. The three sites were Geisinger (GE), an open healthcare system serving central and northeastern Pennsylvania, and two integrated delivery systems, Kaiser Permanente Washington (KPWA) and Kaiser Permanente Mid-Atlantic States (KPMAS), which are closed-network systems serving insured members in Washington state and in Washington D.C., Maryland, and Virginia, respectively. All sites have existing infrastructure through established genetic counseling programs, described in greater detail in Dinucci et al. 2021 [11].

We previously assessed the reach, fidelity, effectiveness, and acceptability of the pilot implementation study of the Traceback programs using quantitative and qualitative methods [21]. The objective of this manuscript is to evaluate the cost, efficiency and performance of implementing a Traceback cascade testing program across three large healthcare systems to identify and conduct genetic testing on women with ovarian cancer who were previously untested for inherited ovarian cancer syndromes and to facilitate cascade testing of their at-risk relatives. Additionally, we generalize our findings to report the cost, efficiency, and performance of two hypothetical programs based on our implementation using national estimates.

## 2. Materials and Methods

Implementation—proband identification: Implementation processes for GE and KPWA were similar in nature with respect to proband identification. In GE and KPWA, genetic counseling program staff reached out to the identified probands via letter and invited them to attend a genetic counseling session where they were then offered genetic testing (Supplementary Materials Figures S1 and S2). KPMAS also outreached to identified probands via letter and then followed a different implementation process: a nurse coordinator contacted women who were previously untested and offered a mail-in genetic testing kit sent directly to their homes. Genetic counseling prior to testing was available if requested or required (Supplementary Materials Figure S3). All probands were tested using the Invitae Common Hereditary Cancer Panel, which included 48 genes.

Implementation—cascade testing: Cascade testing processes with respect to the degree of contact with the proband and/or at-risk relatives varied across the three sites for patients with positive genetic testing results. GE discussed cascade testing for at-risk family members with the proband during result disclosure and provided an educational packet and family letters (including enough copies for all identified first-degree relatives) to assist probands in communicating the result with at-risk family members. No other contact was made with the proband following result disclosure. KPMAS also discussed testing for at-risk family members during the proband result disclosure and offered a letter to give family members that described the need for testing. If the proband requested support speaking with their relatives about cascade testing, KPMAS staff could speak with a non-Kaiser Permanente relative only if the proband was also on the call; there was no outreach directly to relatives. KPWA contacted the proband following result disclosure to ask if they had discussed results with at-risk relatives and offered to help the proband facilitate cascade screening, including offering direct contact with relatives on behalf of consenting probands. KPWA could speak to relatives including non-Kaiser Permanente members but could only order genetic testing if the relative was a Kaiser Permanente member.

Performance, cost and efficiency analysis: We developed three decision-analytic models to represent the FACTS implementation programs at three sites. We estimated the direct program implementation costs and efficiency, defined as cost per proband tested and cost per hereditary cancer panel case detected, and evaluated the performance of each implementation program at each of the three sites based on real-world data from the FACTS implementation program results from the perspective of the healthcare system.

Process maps were developed and corresponding activities and sub-activities were defined for each program (Supplementary Materials Figures S1–S3 and Tables S1 and S2) by key program personnel and reviewed by all program implementation team members. The number of patients at each process step and patient uptake behavior was recorded. We applied a bottom-up micro-costing approach to calculate costs by combining additional program-specific resource use with the unit costs of direct cost components based on program activities [22,23]. We included preimplementation costs relating to the set-up and development of program processes and materials, as well as program implementation costs. For healthcare systems with an established genetic counseling program in place, we excluded overhead costs and costs related solely to research purposes, consistent with established costing methodology indicating that infrastructure costs may reasonably be excluded from program-specific cost analysis when implemented within existing capacity [24].

Fixed costs included materials, supplies, and software and labor costs for program development, administration and set-up. Variable costs were patient-specific, varying with the number of patients to whom services were provided [25], and included personnel time, materials and supplies, and genetic testing. We accounted for slight process differences in the result disclosure of positive, VUS, and negative results, both between sites and by disclosure type, which reflected differences in personnel time and material and supply costs.

The time period for GE was between December 2021 and March 2022 for preimplementation, with active implementation (defined as first patient outreach to last phone call date) occurring between March 2022 and February 2023. The time period for KPWA and KPMAS was between May 2021 and June 2022 for preimplementation, with active implementation occurring between June 2022 and May 2023. Costs were reported in 2023 U.S. dollars and adjusted with the Consumer Price Index (CPI) [26].

Generalizability analysis: We conducted additional analysis to estimate potential generalizable outcomes using national cost estimates, uptake observations from FACTS programs, estimates from the literature, and expert opinion. We developed two models based on the program structure and processes from KPWA and KPMAS; we excluded GE given the high degree of similarity between the GE and KPWA programs with respect to the proband and to include the KPWA approach of offering direct contact with respect to cascade testing. We also conducted four scenario analyses based on the generalizable models: (1) assuming 100% uptake for all behavioral parameters (Supplementary Materials Table S5), (2) varying costs at 80% and 120% of base-case values, (3) varying personnel time at 80% and 120% of base-case values, and (4) varying costs and personnel time simultaneously at 80% and 120% of base-case values (Supplementary Materials Tables S9–S11). In the generalizability analysis, representative personnel positions and time for specific sub-activity tasks were determined based on expert panel input comprised of program leaders/genetic counselors from the three programs and data from the Traceback programs. Salary costs were obtained from the Bureau of Labor Statistics, and we assumed a 20% fringe benefit rate [27,28,29,30,31,32]. Representative personnel positions in the generalizable models included a genetic counselor, licensed practical nurse, and medical assistant. Genetic testing costs were based on Medicare reimbursement rates [33]. Parameter estimates were derived from multiple sources, including the literature, observed program data, and expert opinion, depending on the parameter. For example, the percentage of patients previously untested was obtained from the literature, while the percentage of patients returning the genetic testing panel kit was based on program data and expert opinion (Supplementary Materials Tables S6–S8).

Data sources: Standardized comprehensive data collection instruments were developed to retrospectively collect data based on each of the program activities and sub-activity tasks and completed by applicable program personnel. Implementation data was obtained through multiple sources. The initial study population was identified through an electronic health record (EHR) data pull at each site, and eligibility was determined through chart review. Personnel position, hourly salary (including fringe benefits) and time data were provided by program staff. Material and supply costs were obtained from invoices from financial records, and patient mailings were based on United States Postal Service postage rates [34]. Genetic testing costs were based on financial records (GE) and Medicare reimbursement rates for KPWA and KPMAS. Performance and uptake data including the number of patients at each step and genetic testing results were obtained from program-specific databases (Table 1) with additional detail on process performance outcomes in Supplementary Materials Tables S3 and S4.

## 3. Results

### 3.1. Performance

#### 3.1.1. Proband Testing

The initial study population consisted of 450, 240, and 878 living patients with ovarian cancer identified at GE, KPWA, and KPMAS, respectively. Of those, 224 (49.8%) were previously untested, i.e., eligible for genetic testing in at GE, 149 (62.1%) at KPWA, and 224 (25.5%) at KPMAS. Among eligible patients, 129 (57.6%), 72 (48.3%), and 153 (68.3%) were reached in GE, KPWA, and KPMAS, respectively, and 33 (14.7%) and 41 (27.5%) were reached and scheduled genetic counseling appointments in GE and KPWA. Of eligible patients at KPMAS, 123 (54.9%) were reached and consented to genetic testing (without genetic counseling), and two (0.9%) requested or required genetic counseling; both attended genetic counseling. Of the 123 patients who consented at KPMAS, 74 (60.2%) returned their mail-in genetic testing kit and underwent genetic testing; 49 (39.8%) consented but did not return the kit. In GE and KPWA, 30 (13.4%) and 41 (27.5%) of eligible patients, respectively, attended a genetic counseling appointment (Table 1).

Among patients eligible for testing, 18 (8.0%), 37 (24.8%), and 74 (33.0%) underwent genetic testing in GE, KPWA, and KPMAS, respectively. At GE, one (0.4%) patient had a positive genetic test result for another cancer risk gene (not BRCA), five (2.2%) had a variant of unknown significance (VUS) result, and 12 (5.4%) had a negative result. In KPWA, there were two (1.3%) patients with a positive genetic test result (both other cancer risk genes), 10 (6.7%) with a VUS result and 25 (16.8%) with a negative result. In KPMAS, seven (3.1%) patients had a positive genetic test result (three (1.3%) for *BRCA* and four (1.8%) other cancer risk genes), 21 (9.4%) had a VUS result, and 46 (20.5%) had a negative result (Table 1).

#### 3.1.2. Cascade Testing

At KPWA, 21 at-risk relatives were identified from patients with a positive genetic test result. None of the at-risk relatives identified had Kaiser Permanente insurance and none received genetic testing through the program (Table 1).

### 3.2. Cost and Efficiency

Table 2 summarizes program cost and efficiency outcomes by site. Total program cost for GE was $41,564, consisting of $20,424 in fixed costs and $21,139 in variable costs. KPWA program costs totaled $97,396, of which $29,403 were fixed costs and $67,993 were variable costs. KPMAS program cost was $120,327 in total, with $26,751 in fixed costs and $93,575 in variable costs (Table 2).

Greater operational efficiency was observed at KPMAS compared to the other two sites, with the lowest cost per patient tested ($1626) and the lowest cost per hereditary cancer panel case detected ($17,190). GE had a cost per patient tested of $2309 and a cost per hereditary cancer panel case detected of $41,564, similar to KPWA, which had a cost per patient tested of $2632 and a cost per hereditary cancer panel case detected of $48,698 (Table 2).

In terms of the cost breakdown by category, personnel costs were comparable across GE and KPWA and comprised almost 60% of total program costs, with genetic testing costs accounting for slightly over 40% of total costs. In KPMAS, genetic testing comprised the majority (almost 70%) of the total costs, with personnel costs accounting for almost 30% (Table 3).

### 3.3. Generalizable Model

#### 3.3.1. Performance

Based on the generalizable model assuming 300 living ovarian cancer patients identified at a hypothetical Traceback program, we estimate 180 (60%) as previously untested, i.e., eligible for genetic testing, of which 71 (39.7%) would undergo genetic testing in a Traceback program similar to the KPWA program and 50 (28.0%) in a Traceback program similar to KPMAS. Under a KPWA-like program approach, we estimate 11 (6.0%) probands to have positive genetic testing panel results and approximately 43 at-risk relatives identified, of which 15 (34.0%) undergo genetic testing and seven (17.0%) test positive for a hereditary cancer panel gene. Under a KPMAS-like program approach, we estimate eight (4.2%) probands to have positive genetic testing panel results and approximately 30 at-risk relatives identified, of which nine (30.0%) undergo genetic testing and five (15.0%) test positive for a hereditary cancer panel gene (Table 4).

#### 3.3.2. Cost and Efficiency

The total program cost for a potential KPWA program was estimated to be $118,177 compared to a total cost of $88,106 for a KPMAS program, with fixed and variable cost breakdowns shown in Table 5. Efficiency in terms of cost per patient tested ($1374 and $1480) and cost per hereditary cancer panel case detected ($6564 and $7275) was similar across both KPWA and KPMAS programs, respectively. We conducted a scenario analysis assuming 100% uptake for all patient behavioral parameters, which showed that efficiency results were also similarly comparable across both programs (Table 5).

**Table 5:** Generalizability cost and efficiency outcomes.

	Base Case		Scenario 100% Uptake	
	KPWA	KPMAS	KPWA	KPMAS
**Costs**				
Total Costs	$118,177	$88,106	$291,902	$279,515
Variable Costs	$97,277	$67,206	$271,002	$258,615
Fixed Costs	$20,900	$20,900	$20,900	$20,900
**Efficiency**				
Cost per patient tested	$1374	$1480	$1014	$971
Cost per hereditary cancer panel case detected	$6564	$7275	$3604	$3451

Note: Costs are in 2023 USD. KPWA: Kaiser Permanente Washington; KPMAS: Kaiser Permanente Mid-Atlantic States.

## 4. Discussion

We compared three Traceback programs with different implementations across sites. All sites used similar approaches to proband identification but differed in their genetic testing approaches: Geisinger and KPW referred probands for genetic counseling, while the KPMAS nurse coordinator offered probands genetic testing directly. Each of the three programs had different levels of contact with proband relatives: no contact for GE; phone contact only if the relative joined the proband’s call for KPMAS; and KPWA offered direct contact if the proband consented. Our previous work demonstrated the feasibility, reach and acceptability of the pilot programs [21]; the present analysis expands on those findings to evaluate cost and efficiency, adding early foundational data on program implementation costs. Since Traceback programs are relatively new in U.S. settings, the costs of implementing Traceback programs are unknown. Thus, direct cost comparison with other studies is challenging: traditional cost-effectiveness outcomes such as the incremental cost–utility ratio reported by Moya-Alarcón et al. 2019 or the incremental cost-effectiveness ratio reported by Eccleston et al. 2017 are not comparable, as they are not based on program implementation costs [15,16]. We estimated total direct costs including both fixed and variable costs, which may inform decision-makers in healthcare systems considering Traceback programs.

The KPMAS Traceback program had higher rates of genetic testing, where a third of eligible patients underwent genetic testing compared to one fourth in KPWA and about 8% in GE. This difference may reflect the streamlined process at KPMAS, where a nurse offered testing over the phone and mailed genetic testing kits to the individual—as opposed to requiring a genetic counseling appointment prior to testing. This is consistent with emerging evidence of higher testing uptake associated with mainstreaming of genetic testing services [35]. However, given the observational and non-randomized design of this pilot implementation, this should not be interpreted as a causal effect of the program design. To date, there is one other United States-based study measuring the feasibility of the Traceback methodology in ovarian cancer survivors. White et al. report a genetic testing uptake of 29%, which is in line with our findings [36]. Direct contact, which was offered at KPWA, is associated with higher cascade genetic testing rates than patient-mediated approaches [37], although no at-risk relatives obtained testing through the program.

Fixed costs represented program preimplementation costs and mainly comprised the cost of materials and supplies and start-up activities including data pulls to identify living ovarian cancer patients and the development of outreach materials. Absolute fixed costs were comparable and relatively in line across the three sites, ranging from $20,424 to $29,403. Total direct program costs were mainly driven by variable costs from genetic testing patient volume. KPMAS had the highest variable cost explained by the higher number of patients undergoing genetic testing: 74 patients compared to 37 patients in KPWA and 18 patients in GE. While GE had the lowest personnel costs in absolute terms and per eligible patient, KPMAS personnel costs per patient tested ($472) were substantially lower than those at GE ($1350) and KPWA ($1505). This difference is again attributable to the program design forgoing genetic counseling unless requested by the patient prior to testing, as well as to higher genetic testing volume and uptake. Higher personnel position levels performing program activities and longer time estimates explain the higher costs per patient tested observed in KPWA.

For generalizability, we modeled the performance, cost and efficiency of two hypothetical Traceback programs based on the KPWA and KPMAS Traceback program processes. The KPWA hypothetical program performed better in terms of uptake of genetic testing, with 81% of those reached and scheduled genetic counseling undergoing genetic testing in the base-case compared to KPMAS, where 57% of those who consented to genetic testing underwent genetic testing. While the KPMAS program process was more direct in proband testing, with applied uptake parameters aligned at the front end of the process across the two programs, the lower genetic testing uptake in KPMAS in comparison to KPWA was explained by loss to follow-up at the mail-in kit return step, which is specific to the KPMAS process. The KPWA program also performed better in cascade testing due to accounting for the positive impact of direct contact in the model parameters. Overall, efficiency was comparable between the two programs because total costs were lower in the KPMAS process due to lower uptake of genetic testing, but a higher number of hereditary breast and ovarian cases were detected in the KPWA process. This is assuming that 100% uptake for behavioral parameters translates to an equal number of cases detected but slightly lower overall costs under the KPMAS approach as expected due to minimized pre-testing genetic counseling costs.

We note several limitations. First, all three sites have well-established genetic counseling programs, which may not be generalizable to other healthcare systems. The generalizable models are also based on the assumption that genetic counseling infrastructure is already in place, which may make them less applicable to systems without such infrastructure. Personnel costs were also impacted by the level of personnel performing the tasks; however, we demonstrated a range of possibilities using both real-world data and appropriate standard position levels in the generalizability analysis taking into account program leaders’ input. Some personnel time estimates on specific sub-activities were based on retrospective recollection, which may be subject to recall bias. To address this and related cost uncertainty, we conducted additional scenario analyses varying costs and personnel time independently and jointly by ±20% of base-case values in the generalizable models. Data on uptake of cascade testing was limited due to only one of the programs identifying at-risk relatives and limitations within the Kaiser Permanente system around the ability to talk with relatives and/or order genetic testing if the relative was not insured by Kaiser Permanente. Although the study team partnered with a testing vendor to obtain counts of relatives who accessed testing through their family referral program, it is possible that relatives completed testing with another testing vendor, which we were unable to examine here. This represents a critical gap in pilot evaluation and an important real-world finding about system-level barriers to cascade testing in closed-network health systems. While open-network systems may have advantages with respect to authorization barriers, testing completed with an outside provider, system, or geographic location presents data challenges to both open and closed systems. Additional data and methodological approaches, such as proband follow-up surveys, are needed to fully evaluate cascade testing uptake given the limitations around accessing relatives’ testing data. Future work also includes incorporating sustainability costs, as we would expect a lower annual volume of ovarian cancer patients as programs continue.

## 5. Conclusions

Traceback programs are feasible to implement and can improve the uptake of genetic testing for ovarian cancer patients at costs that healthcare systems may find acceptable. Mail-in genetic testing kits with nurse support were associated with better performance and efficiency compared to requiring pre-testing genetic counseling visits and on-site testing, although this should not be interpreted as a causal effect of program design given the observational, non-randomized design of the pilot implementation. Quantifying the program costs can inform healthcare system decision-makers on resource allocation when considering similar program development and implementation.

## Figures and Tables

**Table 1:** Ovarian cancer Traceback cascade testing program performance outcomes.

	GE		KPWA		KPMAS	
Performance	*N*	(%)	*N*	(%)	*N*	(%)
**Proband Identification Outcomes**						
Number of living ovarian cancer patients identified	450	-	240	-	878	-
Number of ovarian cancer patients who are untested/eligible for genetic testing (out of patients identified)	224	49.8%	149	62.1%	224	25.5%
Number of eligible patients reached (out of eligible patients)	129	57.6%	72	48.3%	153	68.3%
**Proband Genetic Testing Outcomes**	***N*** ** = 224**		***N*** ** = 149**		***N*** ** = 224**	
Number of eligible patients reached and scheduled genetic counseling and testing	33	14.7%	41	27.5%	N/A	N/A
Number of eligible patients reached and consent to genetic testing (without genetic counseling) (KPMAS only)	NA	NA	NA	NA	123	54.9%
Number of eligible patients reached and require or request genetic counseling (KPMAS only)	NA	NA	NA	NA	2	0.9%
Number of eligible patients who attend genetic counseling appointment	30	13.4%	41	27.5%	2	0.9%
Number of patients who undergo genetic testing	18	8.0%	37	24.8%	74	33.0%
Number of patients with genetic testing panel positive result	1	0.4%	2	1.3%	7	3.1%
Number of patients with genetic testing panel positive *BRCA* result	0	0.0%	0	0.0%	3	1.3%
Number of patients with genetic testing panel positive (other cancer risk gene) result	1	0.4%	2	1.3%	4	1.8%
Number of patients with genetic testing panel result variant of unknown significance	5	2.2%	10	6.7%	21	9.4%
Number of patients with genetic testing panel result negative	12	5.4%	25	16.8%	46	20.5%
**At-Risk Relative Identification for Cascade Testing Outcomes**						
Total number of at-risk relatives identified	NA	-	21	-	NA	-
Number of at-risk relatives identified who attend genetic counseling appointment	NA	NA	0.0	0.0%	NA	NA
Total number of at-risk relatives identified who undergo genetic testing	NA	NA	0.0 *	0.0%	NA	NA

* None of the at-risk relatives identified were system members and none received genetic testing through the program. Genetic testing data from one lab (Invitae) showed no at-risk relatives received genetic testing. GE: Geisinger; KPWA: Kaiser Permanente Washington; KPMAS: Kaiser Permanente Mid-Atlantic States. NA: Not Applicable.

**Table 2:** Ovarian cancer Traceback cascade testing program cost and efficiency outcomes.

	GE	KPWA	KPMAS
**Costs**			
Total Costs	$41,564	$97,396	$120,327
Variable Costs	$21,139	$67,993	$93,575
Fixed Costs	$20,424	$29,403	$26,751
**Efficiency**			
Cost per patient tested	$2309	$2632	$1626
Cost per hereditary cancer panel case detected	$41,564	$48,698	$17,190

Note: Costs are in 2023 USD. GE: Geisinger; KPWA: Kaiser Permanente Washington; KPMAS: Kaiser Permanente Mid-Atlantic States.

**Table 3:** Cost breakdown by category.

	GE		KPWA		KPMAS	
Cost Category	Cost	(%)	Cost	(%)	Cost	(%)
**Total Costs**	**$41,564**	**-**	**$97,396**	-	$120,327	-
Personnel Costs	$24,309	58.5%	$55,702	57.2%	$34,908	29.0%
Material and Supply Costs	$155	0.4%	$253	0.3%	$2539	2.1%
Genetic Testing Costs	$17,100	41.1%	$41,440	42.5%	$82,880	68.9%
**Variable Costs**	**$21,139**	**-**	**$67,993**	**-**	**$93,575**	**-**
Personnel Costs	$3884	18.4%	$26,300	38.7%	$10,655	11.4%
Material and Supply Costs	$155	0.7%	$253	0.4%	$41	0.0%
Genetic Testing Costs	$17,100	80.9%	$41,440	60.9%	$82,880	88.6%
**Fixed Costs**	**$20,424**	**-**	**$29,403**	**-**	**$26,751**	**-**
Personnel Costs	$20,424	100.0%	$29,403	100.0%	$24,253	90.7%
Material and Supply Costs	$0	0.0%	$0	0.0%	$2498	9.3%

Note: Costs are in 2023 USD. GE: Geisinger; KPWA: Kaiser Permanente Washington; KPMAS: Kaiser Permanente Mid-Atlantic States.

**Table 4:** Generalizability performance outcomes (base case and scenario analysis).

	Base-Case				Scenario			
	KPWA	KPWA	KPMAS	KPMAS	KPWA	KPWA	KPMAS	KPMAS
Performance	*N*	(%)	*N*	(%)	*N*	(%)	*N*	(%)
**Proband Identification Outcomes**								
Number of living ovarian cancer patients identified through data pull for Ovarian Cancer Traceback Cascade Testing Program	300	-	300	-	300	-	300	-
Number of ovarian cancer patients who are untested/eligible for genetic testing (% of patients identified) (out of patients identified)	180	60.0%	180	60.0%	180	60.0%	180	60.0%
Number of eligible patients reached (out of eligible patients)	126	70.0%	126	70.0%	180	100.0%	180	100.0%
**Proband Genetic Testing Outcomes**	**(** ***N*** ** = 180)**							
Number of eligible patients reached and scheduled genetic counseling/testing	88	49.0%	NA	NA	180	100.0%	NA	NA
Number of eligible patients reached and consent to genetic testing (without genetic counseling) (KPMAS only)	NA	NA	76	42.0%	NA	NA	180	100.0%
Number of eligible patients reached and require or request genetic counseling (KPMAS only)	NA	NA	13	7.0%	NA	NA	0	0.0%
Number of eligible patients who attend genetic counseling appointment	79	44.1%	11	6.3%	180	100.0%	0	0.0%
Number of patients who undergo genetic testing	71	39.7%	50	28.0%	180	100.0%	180	100.0%
Number of patients with genetic testing panel positive result	11	6.0%	8	4.2%	27	15.0%	27	15.0%
Number of patients with genetic testing panel result negative	61	33.7%	43	23.8%	153	85.0%	153	85.0%
**At-Risk Relative Identification for Cascade Testing Outcomes**								
Total number of at-risk relatives identified	43	-	30	-	108	-	108	-
Total number of at-risk relatives identified who attend genetic counseling appointment	15	34.0%	9	30.0%	108	100.0%	108	100.0%
Total number of at-risk relatives identified who undergo genetic testing	15	34.0%	9	30.0%	108	100.0%	108	100.0%
Total number of at-risk relatives identified with positive result	7	17.0%	5	15.0%	54	50.0%	54	50.0%
Total number of at-risk relatives identified with negative result	7	17.0%	5	15.0%	54	50.0%	54	50.0%

KPWA: Kaiser Permanente Washington; KPMAS: Kaiser Permanente Mid-Atlantic States. NA: Not Applicable.

## Data Availability

Data are available on request due to restrictions (e.g., privacy, legal or ethical reasons).

## Supplementary Materials

The following supporting information can be downloaded at https://www.mdpi.com/article/10.3390/cancers18142292/s1, Figure S1. Geisinger Ovarian Cancer Traceback Cascade Testing Program; Figure S2. Kaiser Permanente Washington Ovarian Cancer Traceback Cascade Testing Program; Figure S3. Kaiser Permanente Mid-Atlantic States Ovarian Cancer Traceback Cascade Testing Program; Table S1. Program Activities and Sub-activities (GE and KPWA); Table S2. Program Activities and Sub-activities (KPMAS); Table S3. Performance Outcomes (GE and KPWA); Table S4. Performance Outcomes (KPMAS); Table S5. Generalizability Input Parameters (KPWA); Table S6. Generalizability Uptake Parameters (KPWA); Table S7. Generalizability Input Parameters (KPMAS); Table S8. Generalizability Uptake Parameters (KPMAS); Table S9. Scenario Analysis on Costs as a Percentage of Base-Case Costs; Table S10. Scenario Analysis on Personnel Time as a Percentage of Base-Case Personnel Time; Table S11. Scenario Analysis on Costs and Personnel Time as a Percentage of Base-Case Costs and Base-Case Personnel Time. Refs. [38,39,40,41,42,43,44,45,46,47] are cited in Supplementary Materials.
