# Supplementary material for: Evaluation of Costs, Efficiency and Performance of a Pilot Genetic Testing Traceback Program for Ovarian Cancer
Source: Cancers (Basel). 2026 Jul 16;18(14):2292. doi: 10.3390/cancers18142292 (PMC13406528; doi:10.3390/cancers18142292)
Supplement: Supplementary file 1 [file cancers-18-02292-s001.zip › cancers-4379420-supplementary.pdf]

Supplementary Materials

Figure S1. **Geisinger ovarian cancer Traceback cascade testing program.**

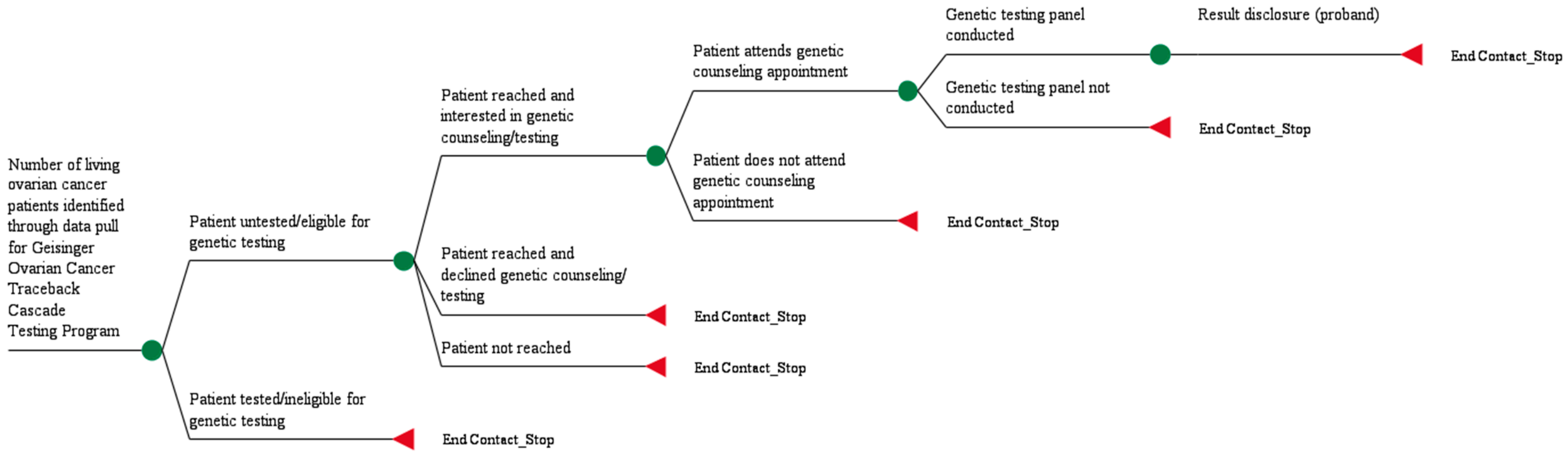

Figure S2. Kaiser Permanente Washington ovarian cancer Traceback cascade testing program.

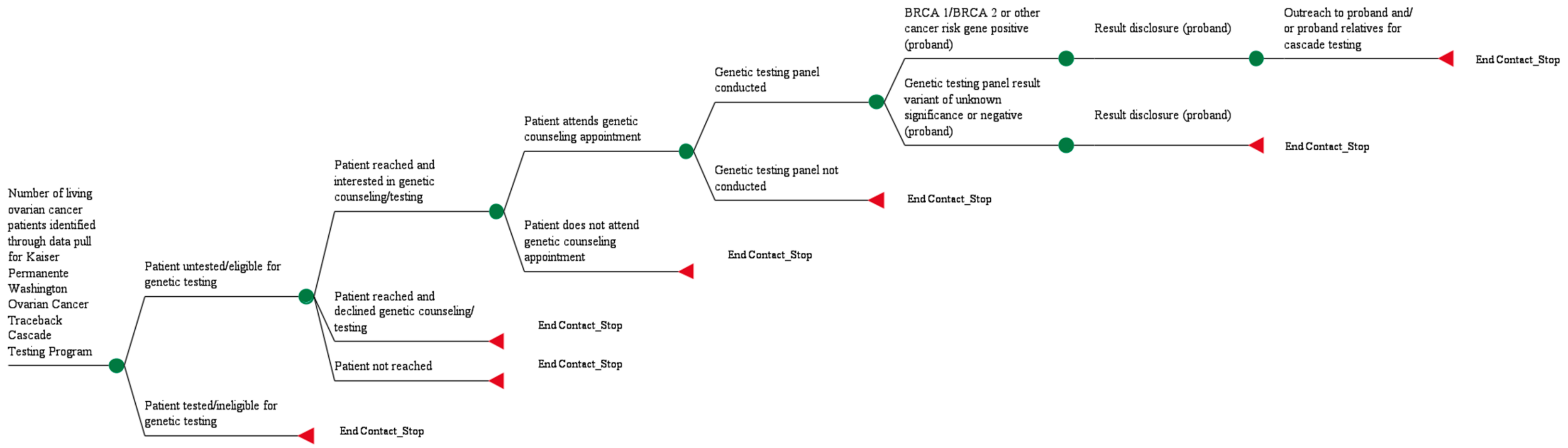

Figure S3. **Kaiser Permanente Mid-Atlantic States ovarian cancer Traceback cascade testing program.**

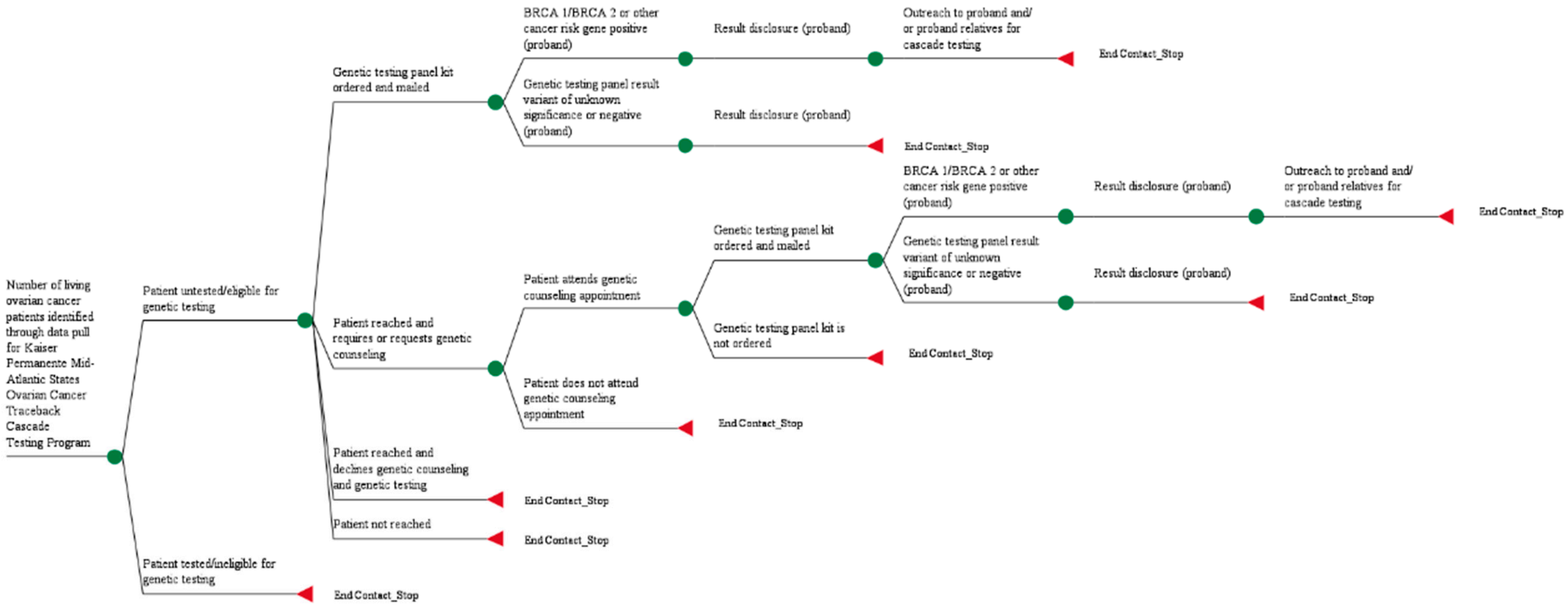

Table S1. Program activities and sub-activities (GE and KPWA).

**Preimplementation Activities**	**Sub-activities**
Initial data pull	• Personnel identify and prepare codes for data pull<br>• Personnel conduct data pull and clean the data to identify the number of living ovarian cancer patients
Patient outreach materials	• Personnel develop patient outreach materials
**Implementation Activities**	**Sub-activities**
Chart review conducted to determine patient eligibility	• Personnel conduct chart review on living ovarian cancer patients to identify those untested and eligible for genetic testing
Patient outreach prioritized	• Personnel review eligible patients and identify those with and without appointments with a system provider to prioritize outreach
Patient invitation letters and informational material sent	• Personnel send patient invitation letters and informational material (by mail and/or through patient portal) to eligible patients
Follow-up conducted	• Personnel conduct up to 3 follow-up phone calls with eligible patients
Genetic counseling appointment scheduled	• Personnel place genetic counseling referral and schedule genetic counseling appointment for eligible patients reached and interested in genetic counseling/testing
Genetic counseling appointment reminder	• Personnel conduct phone call reminder of genetic counseling appointment for eligible patients reached and interested in genetic counseling/testing<br>• Personnel may or may not include offer to collect family history for pedigree for eligible patients reached and interested in genetic counseling/testing
Genetic testing panel conducted	• Patient undergoes genetic testing
Result disclosure (proband positive)	• Personnel disclose genetic testing results and review family pedigree to recommend which family members would benefit from pursuing cascade testing to patients with a positive result via phone call or telehealth visit<br>• Personnel send genetic testing result disclosure letters (and family letters if applicable) by mail and/or through patient portal
Result disclosure (proband variant of unknown significance)	• Personnel disclose genetic testing results via phone call or telehealth visit, and/or through the patient portal, and/or by mail
Result disclosure (proband negative)	• Personnel disclose genetic testing results via phone call or telehealth visit, and/or through the patient portal, and/or by mail
Outreach to proband and/or proband relatives for cascade testing	• Personnel time to reach out to proband to follow-up on discussions with relatives for cascade testing

GE: Geisinger; KPWA: Kaiser Permanente Washington.

Table S2. Program activities and sub-activities (KPMAS).

**Preimplementation Activities**	**Sub-activities**
Initial data pull	• Personnel identify and prepare codes for data pull<br>• Personnel conduct data pull and clean the data to identify the number of living ovarian cancer patients
Patient outreach materials	• Personnel develop patient outreach materials
**Implementation Activities**	**Sub-activities**
Chart review conducted to determine patient eligibility	• Personnel conduct chart review on living ovarian cancer patients to identify those untested and eligible for genetic testing
Patient invitation letters and informational material sent	• Personnel send patient invitation letters and informational material (by mail and/or through patient portal) to eligible patients
Follow-up phone calls conducted	• Personnel conduct up to 2 follow-up phone calls with eligible patients
Genetic testing panel kit ordered and mailed	• Personnel place genetic testing order for genetic testing panel kit to be mailed for all those who consented to genetic testing (without genetic counseling)
Genetic testing panel conducted	• Patient undergoes genetic testing
Reminder to return genetic testing panel kit	• Personnel conduct phone call to remind patients to return genetic testing kit if it has not been returned within 30 days of kit order date
Genetic counseling appointment scheduled	• Personnel place genetic counseling referral and schedule genetic counseling appointment for eligible patients reached who require or request genetic counseling
Patient attends genetic counseling appointment	• Personnel conduct genetic counseling via phone call or telehealth visit including ordering genetic testing kit
Genetic testing panel kit ordered and mailed (after genetic counseling)	• Personnel place genetic testing order for genetic testing panel kit to be mailed for all those who consented to genetic testing (after genetic counseling)
Patient informed that test results are available and genetic counseling appointment for result disclosure scheduled if applicable	• Personnel conduct phone call to inform patients that test results are available for patients with positive, variant of unknown significance, or negative results<br>• Personnel schedule genetic counseling visit for patients with a positive result
Result disclosure (proband positive)	• Personnel disclose genetic testing results to patients with a positive result and review family pedigree to recommend which family members would benefit from pursuing cascade testing via phone call or telehealth visit<br>• Personnel send genetic testing result disclosure letters by mail and/or through patient portal
Result disclosure (proband variant of unknown significance)	• Personnel disclose genetic testing results through the patient portal or by mail if the patient does not have a patient portal account
Result disclosure (proband negative)	• Personnel disclose genetic testing results through the patient portal or by mail if the patient does not have a patient portal account
Outreach to proband for cascade testing	• Personnel time to reach out to proband to follow-up on discussions with relatives for cascade testing

KPMAS: Kaiser Permanente Mid-Atlantic States.

Table S3. Performance outcomes (GE and KPWA).

Performance Outcome	GE	KPWA
Number of living ovarian cancer patients identified	450	240
Percentage of living ovarian cancer patients previously untested/eligible for genetic testing	50%	62%
Percentage of eligible patients that are sent invitation letters and informational material through the patient portal	45%	NA
Percentage of eligible patients that are mailed invitation letters and informational material	100%	100%
Percentage of eligible patients reached and interested in genetic counseling/testing	15%	28%
Percentage of eligible patients reached and decline genetic counseling/testing	43%	21%
Percentage of patients that attend genetic counseling appointment among those reached and interested in genetic counseling/testing	91%	100%
Percentage of eligible patients who undergo genetic testing among those reached and interested in genetic counseling/testing who attended genetic counseling appointment	60%	90%
Percentage of genetic testing panel result positive among eligible patients who underwent genetic testing (proband)	6%	5%
Percentage of genetic testing panel result variant of uncertain significance among eligible patients who underwent genetic testing (proband)	28%	27%
Percentage of patients with a positive result that are reached via phone call or telehealth visit for result disclosure	100%	100%
Percentage of patients with a positive result that are sent result disclosure letters (and family letters if applicable) through the patient portal	100%	100%
Percentage of patients with a positive result that are sent result disclosure letters (and family letters if applicable) through the mail	100%	NA
Percentage of patients with a variant of unknown significance result that are reached via phone call or telehealth visit for result disclosure	100%	100
Percentage of patients with a variant of unknown significance result that are sent result disclosure letters (and family letters if applicable) through the patient portal	100%	100%
Percentage of patients with a variant of unknown significance result that are sent result disclosure letters (and family letters if applicable) through the mail	100%	NA
Percentage of patients with a negative result that are reached via phone call or telehealth visit for result disclosure	100%	NA
Percentage of patients with a negative result that are sent result disclosure letters (and family letters if applicable) through the patient portal	NA	100
Percentage of patients with a negative result that are sent result disclosure letters (and family letters if applicable) through the mail	0%	0%

GE: Geisinger; KPWA: Kaiser Permanente Washington; NA: Not Applicable.

Table S4. Performance outcomes (KPMAS).

Performance Outcome	KPMAS
Number of living ovarian cancer patients identified	878
Percentage of living ovarian cancer patients previously untested/eligible for genetic testing	26%
Percentage of eligible patients that are sent invitation letters and informational material through the patient portal	92%
Percentage of eligible patients reached and consent to genetic testing	55%
Percentage of eligible patients reached and require or request genetic counseling	1%
Percentage of eligible patients reached and decline genetic counseling and genetic testing	13%
Percentage of eligible patients who consent to genetic testing who return genetic testing panel kit within 30 days of kit order date	50%
Percentage of eligible patients who return genetic testing panel kit following reminder phone call among those that consented to genetic testing and did not return kit within 30 days of kit order date	21%
Percentage of genetic testing panel result positive among eligible patients who underwent genetic testing (for patients who did not have genetic counseling)	9%
Percentage of genetic testing panel result variant of uncertain significance among eligible patients who underwent genetic testing (for patients who did not have genetic counseling)	28%
Percentage of *BRCA* 1 or *BRCA* 2 positive result out of genetic testing panel result positive (for patients who did not have genetic counseling)	43%
Percentage of patients that attend genetic counseling appointment	100%
Percentage of patients with a positive result that are reached via phone call or telehealth visit for result disclosure	71%
Percentage of patients with a positive result that are sent result disclosure letters through the patient portal	86%
Percentage of patients with a positive result that are sent result disclosure letters through the mail	14%
Percentage of patients with a positive result that are not reached via phone or telehealth visit for result disclosure	29%
Percentage of patients with a positive result that remain unreached after personnel phone call attempts	100%
Percentage of patients with a variant of unknown significance result that are sent result disclosure letters through the patient portal	95%
Percentage of patients with a variant of unknown significance result that are sent result disclosure letters through the mail	5%
Percentage of patients with a negative result that are sent result disclosure letters through the patient portal	98%
Percentage of patients with a negative result that are sent result disclosure letters through the mail	2%

KPMAS: Kaiser Permanente Mid-Atlantic States.

Table S5. Generalizability input parameters (KPWA).

**Fixed Cost Components**					
**Preimplementation Activities**	**Sub-activities**	**Personnel Time (hours)**	**Personnel Time (hours)**	**Materials and Supplies Costs ($)**	
Materials and Supplies					
Initial data pull	• Personnel identify and prepare codes for data pull<br>• Personnel conduct data pull and clean the data to identify the number of living ovarian cancer patients	Data Scientist			
Patient outreach materials	• Personnel develop patient outreach materials	Project Management Specialist;<br>Genetic Counselor;<br>Physician	30.00<br>5.00<br>2.00		
**Variable Cost Components**					
**Implementation Activities**	**Sub-activities**	**Personnel Position**	**Personnel Time (minutes/patient)**	**Materials and Supplies Cost ($/patient)**	**Genetic Testing Cost ($/patient)**
Chart review conducted to determine patient eligibility	• Personnel conduct chart review on living ovarian cancer patients to identify those untested and eligible for genetic testing	Genetic Counselor	10.00		
Patient invitation letters and informational material sent	• Personnel send patient invitation letters and informational material (by mail and/or through patient portal) to eligible patients	Medical Assistant	7.00*	$1.50	
Follow-up conducted	• Personnel conduct up to 3 follow-up phone calls with eligible patients	Medical Assistant	15.00		
Genetic counseling appointment scheduled	• Personnel place genetic counseling referral and schedule genetic counseling appointment for eligible patients reached and interested in genetic counseling/testing	Medical Assistant	10.00		
Patient attends genetic counseling appointment	• Patient attends genetic counseling appointment and personnel conduct genetic counseling	Genetic Counselor	60.00		

Genetic testing panel conducted	• Percentage of eligible patients who undergo genetic testing among those reached and interested in genetic counseling/testing who attended genetic counseling appointment (%)				$1,120.00
Result disclosure (proband positive)	• Personnel disclose genetic testing results and review family pedigree to recommend which family members would benefit from pursuing cascade testing to patients with a positive result via phone call or telehealth visit<br>• Personnel send genetic testing result disclosure letters (and family letters if applicable) by mail and/or through patient portal	Genetic Counselor	45.00†	$1.50	
Result disclosure (proband variant of unknown significance)	• Personnel disclose genetic testing results via phone call or telehealth visit, and/or through the patient portal, and/or by mail	Genetic Counselor	35.00†	$1.50	
Result disclosure (proband negative)	• Personnel disclose genetic testing results via phone call or telehealth visit, and/or through the patient portal, and/or by mail	Genetic Counselor	20.00†	$1.50	
**Cascade Testing**					
Outreach to proband and/or proband relatives for cascade testing	• Personnel time to reach out to proband to follow-up on discussions with relatives for cascade testing	Genetic Counselor	15.00		
Proband relatives attend genetic counseling and genetic testing panel conducted	• Personnel conduct genetic counseling on at-risk proband relatives identified	Genetic Counselor	45.00		$375.00
Result disclosure (relatives)	• Personnel disclose genetic testing results to patients who underwent cascade testing with a positive or negative result via phone call or telehealth visit, and/or through the patient portal, and/or by mail	Genetic Counselor	32.50	$1.50	

Note: Hourly salary rates were based on the Bureau of Labor Statistics (BLS) and adjusted to 2023 USD using the Consumer Price Index (CPI). Salary plus fringe benefits ($/hour): Data Scientist $62.12, Project Management Specialist $57.25, Genetic Counselor $54.01, Physician $137.64, Medical Assistant $22.97. KPWA: Kaiser Permanente Washington. Patient mailings were based on United States Postal Service postage rates. Genetic testing costs were based on Centers for Medicare & Medicaid Services. Clinical Laboratory Fee Schedule 2023 and expert opinion. Time estimates are based on staff estimates from the FACTS programs and expert opinion.

*Personnel time was reduced by 2 minutes for patients without a patient portal account.

† Personnel time was reduced by 10 minutes for patients without patient portal account.

Table S6. Generalizability uptake parameters (KPWA).

Parameter		Reference
Number of living ovarian cancer patients identified	300	FACTS program data; expert opinion
Percentage of living ovarian cancer patients previously untested/eligible for genetic testing	60%	Kurian 2023 [38]; Childers 2017 [39]
Percentage of eligible patients that are sent invitation letters and informational material through the patient portal	60%	Strawley 2023 [40]
Percentage of eligible patients that are mailed invitation letters and informational material	100%	Assumption
Percentage of eligible patients reached and interested in genetic counseling/testing	49%	FACTS program data, expert opinion based on assumption of 70% of eligible patients reached and 70% of reached eligible patients interested and scheduled for genetic counseling/tested
Percentage of patients that attend genetic counseling appointment	90%	FACTS program data, expert opinion
Percentage of eligible patients who undergo genetic testing among those reached and interested in genetic counseling/testing who attended genetic counseling appointment	90%	FACTS program data, expert opinion
Percentage of genetic testing panel result positive among eligible patients who underwent genetic testing (proband)	15%	Kurian 2019 [38]
Percentage of patients with a positive result that are reached via phone call or telehealth visit for result disclosure	100%	Assumption
Percentage of patients with a positive result that are sent result disclosure letters (and family letters if applicable) through the patient portal	60%	Strawley 2023 [40]
Percentage of patients with a positive result that are sent result disclosure letters (and family letters if applicable) through the mail	100%	Assumption
Percentage of patients with a variant of unknown significance result that are reached via phone call or telehealth visit for result disclosure	100%	Assumption
Percentage of patients with a variant of unknown significance result that are sent result disclosure letters (and family letters if applicable) through the patient portal	60%	Strawley 2023 [40]
Percentage of patients with a variant of unknown significance result that are sent result disclosure letters (and family letters if applicable) through the mail	100%	Assumption
Percentage of patients with a negative result that are reached via phone call or telehealth visit for result disclosure	100%	Assumption
Percentage of patients with a negative result that are sent result disclosure letters (and family letters if applicable) through the patient portal	60%	Strawley 2023 [40]

Percentage of patients with a negative result that are sent result disclosure letters (and family letters if applicable) through the mail	100%	Assumption
Percentage of probands with a positive result who give assent/requests for Personnel to contact relatives and provide contact information for relatives	20%	Henrikson 2025 [41], Zepp 2023 [42], Morgan 2023 [43]
Number of at-risk proband relatives identified per proband	4	Zepp 2023 [42], Frey 2020 [44]
Percentage of at-risk proband relatives identified for probands with positive genetic test panel result who attend genetic counseling and have genetic testing panel conducted	34%	Frey 2022, Henrikson 2025 [41], Griffin 2020 [45], Whitaker 2021 [46], Baroutsou 2021 [47]
Percentage of at-risk proband relatives identified who have a positive genetic test result among at-risk proband relatives who attend genetic counseling and have genetic testing panel conducted and identified from probands with positive genetic test result	50%	Griffin 2020 [45], Baroutsou 2021 [47]
Percentage of patients who underwent cascade testing that are reached via phone call or telehealth visit for result disclosure	100%	Assumption
Percentage of patients who underwent cascade testing that are sent result disclosure letters (and family letters if applicable) through the patient portal	60%	Strawley 2023 [40]
Percentage of patients who underwent cascade testing that are sent result disclosure letters (and family letters if applicable) through the mail	100%	Assumption

KPWA: Kaiser Permanente Washington.

Table S7. Generalizability input parameters (KPMAS).

**Fixed Cost Components**					
**Preimplementation Activities**	**Sub-activities**	**Personnel Time (hours)**	**Personnel Time (hours)**	**Materials and Supplies Costs ($)**	
Initial data pull	• Personnel identify and prepare codes for data pull<br>• Personnel conduct data pull and clean the data to identify the number of living ovarian cancer patients	Data Scientist	300.00		
Patient outreach materials	• Personnel develop patient outreach materials	Project Management Specialist; Genetic Counselor; Physician;	30.00<br>5.00<br>2.00		
**Variable Cost Components**					
**Implementation Activities**	**Sub-activities**	**Personnel Position**	**Personnel Time (minutes/patient)**	**Materials and Supplies Cost ($/patient)**	**Genetic Testing Cost ($/patient)**
Chart review conducted to determine patient eligibility	• Personnel conduct chart review on living ovarian cancer patients to identify those untested and eligible for genetic testing	Genetic Counselor	10		
Patient invitation letters and informational material sent	• Personnel send patient invitation letters and informational material (by mail and/or through patient portal by providing patient MRNs and corresponding documents to Documentation and Systems department) to eligible patients	Medical Assistant	7.00*	$1.50	
Follow-up phone calls conducted	• Personnel conduct up to 2 follow-up phone calls with eligible patients	Licensed Practical Nurse	10.00		
Genetic testing panel kit ordered and mailed	• Personnel place genetic testing order for genetic testing panel kit to be mailed for all those who consented to genetic testing (without genetic counseling)	Licensed Practical Nurse, Genetic Counselor	10.00		
Genetic testing panel conducted	• Patient undergo genetic testing				$1,120.00

Reminder to return genetic testing panel kit	• Personnel conduct phone calls to remind patients to return genetic testing kit if it has not been returned within 30 days of kit order date	Licensed Practical Nurse	5.00		
Genetic counseling appointment scheduled	• Personnel places genetic counseling referral and schedules genetic counseling appointment for eligible patients reached who require or request genetic counseling	Licensed Practical Nurse	5.00		
Patient attends genetic counseling appointment	• Personnel conduct genetic counseling via phone call or telehealth visit including ordering genetic testing kit	Genetic Counselor	60.00	$1.50	
Patient informed that test results are available and genetic counseling appointment for result disclosure scheduled if applicable	• Personnel conduct phone calls to inform patients that test results are available for patients with positive, variant of unknown significance, or negative results<br>• Personnel schedule genetic counseling visit for patients with a positive result	Licensed Practical Nurse	7.00		
Result disclosure (proband positive)	• Personnel disclose genetic testing results to patients with a positive result and review family pedigree to recommend which family members would benefit from pursuing cascade testing via phone call or telehealth visit<br>• Personnel send genetic testing result disclosure letters by mail and/or through patient portal	Genetic Counselor	45.00*	$1.50	
Result disclosure (proband variant of unknown significance)	• Personnel disclose genetic testing results through the patient portal or by mail if the patient does not have a patient portal account	Genetic Counselor	15.00	$1.50	
Result disclosure (proband negative)	• Personnel disclose genetic testing results through the patient portal or by mail if the patient does not have a patient portal account	Genetic Counselor	15.00	$1.50	
**Cascade Testing**					
Outreach to proband and/or proband relatives for cascade testing	• Personnel time to reach out to proband to follow-up on discussions with relatives for cascade testing	Licensed Practical Nurse	15.00		

Proband relatives attend genetic counseling and genetic testing panel conducted	• Personnel conduct genetic counseling on at-risk proband relatives identified	Genetic Counselor	45.00		$375.00
Result disclosure (relatives)	• Personnel disclose genetic testing results and reviews family pedigree to recommend which family members would benefit from pursuing cascade testing via phone call or telehealth visit and send genetic testing result disclosure letters by mail and/or through patient portal (relatives)	Genetic Counselor	32.50	$1.50	

Note: Hourly salary rates were based on the Bureau of Labor Statistics (BLS) and adjusted to 2023 USD using the Consumer Price Index (CPI). Salary plus fringe benefits ($/hour): Data Scientist $62.12, Project Management Specialist $57.25, Genetic Counselor $54.01, Physician $137.64, Medical Assistant $22.97, Licensed Practical Nurse $32.78. Patient mailings were based on United States Postal Service postage rates. Genetic testing costs were based on Centers for Medicare & Medicaid Services. Clinical Laboratory Fee Schedule 2023 and expert opinion. Time estimates are based on staff estimates from the FACTS programs and expert opinion. KPMAS: Kaiser Permanente Mid-Atlantic States.

*Personnel time was reduced by 2 minutes for patients without a patient portal account.

† Personnel time was reduced by 10 minutes for patients without patient portal account.

Table S8. Generalizability uptake parameters (KPMAS).

Parameter		Reference
Number of living ovarian cancer patients identified	300	FACTS program data, expert opinion
Percentage of living ovarian cancer patients previously untested/eligible for genetic testing	60%	Kurian 2023 [38]; Childers 2017 [39]
Percentage of eligible patients that are sent invitation letters and informational material through the patient portal	60%	Strawley 2023 [40]
Percentage of eligible patients reached and consent to genetic testing	42%	Expert opinion and program data based on assumption of 0.7 eligible patients reached and 0.7 eligible patients interested and scheduled for genetic counseling/tested
Percentage of eligible patients reached and require or request genetic counseling	7%	Expert opinion and program data based on assumption of 0.7 eligible patients reached and 0.7 eligible patients interested and scheduled for genetic counseling/tested
Percentage of eligible patients who consent to genetic testing who return genetic testing panel kit within 30 days of kit order date	50%	FACTS program data, expert opinion
Percentage of eligible patients who return genetic testing panel kit following reminder phone call among those that consented to genetic testing and did not return kit within 30 days of kit order date	20%	FACTS program data, expert opinion
Percentage of genetic testing panel result positive among eligible patients who underwent genetic testing (for patients who did not have genetic counseling)	15%	Kurian 2019 [38]
Percentage of patients that attend genetic counseling appointment	90%	FACTS program data, expert opinion
Percentage of patients who attend genetic counseling appointment and consent to genetic testing and for whom the genetic testing panel kit is ordered and mailed among eligible patients reached who require or request genetic counseling/testing (%)	90%	FACTS program data, expert opinion
Percentage of eligible patients who consent to genetic testing following genetic counseling who return genetic testing panel kit within 30 days of kit order date	50%	FACTS program data, expert opinion
Percentage of genetic testing panel result positive among eligible patients who underwent genetic testing (for patients who did have genetic counseling)	15%	Kurian 2019 [38]
Percentage of patients with a positive result that are reached via phone call or telehealth visit for result disclosure	100%	Assumption
Percentage of patients with a positive result that are sent result disclosure letters through the patient portal	60%	Strawley 2023 [40]

Percentage of patients with a positive result that are sent result disclosure letters through the mail	100%	Assumption
Percentage of patients with a variant of unknown significance result that are sent result disclosure letters through the patient portal	60%	Strawley 2023 [40]
Percentage of patients with a variant of unknown significance result that are sent result disclosure letters through the mail	100%	Assumption
Percentage of patients with a negative result that are sent result disclosure letters through the patient portal	60%	Strawley 2023 [40]
Percentage of patients with a negative result that are sent result disclosure letters through the mail	100%	Assumption
Number of at-risk proband relatives identified per proband	4	Zepp 2023 [42], Frey 2020 [44]
Percentage of at-risk proband relatives identified for probands with positive genetic test panel result who attend genetic counseling and have genetic testing panel conducted	30%	Griffin 2020 [45], Whitaker 2021 [46]
Percentage of at-risk proband relatives identified who have a positive genetic test result among at-risk proband relatives who attend genetic counseling and have genetic testing panel conducted and identified from probands with positive genetic test result	50%	Griffin 2020 [45], Baroutsou 2021 [47]
Percentage of patients who underwent cascade testing that are reached via phone call or telehealth visit for result disclosure	100%	Assumption
Percentage of patients who underwent cascade testing that are sent result disclosure letters (and family letters if applicable) through the patient portal	60%	Strawley 2023 [40]
Percentage of patients who underwent cascade testing that are sent result disclosure letters (and family letters if applicable) through the mail	100%	Assumption

KPMAS: Kaiser Permanente Mid-Atlantic States.

Table S9. Scenario analysis on costs as a percentage of base-case costs.

	KPWA		KPMAS	
(Percentage of Base-Case Costs)	80%	120%	80%	120%
**Costs**				
**Total Costs**	$94,541	$141,812	$70,493	$105,719
**Ovarian cancer Traceback cascade testing program variable costs**	$77,821	$116,732	$53,773	$80,639
**Ovarian cancer Traceback cascade testing program fixed costs**	$16,720	$25,080	$16,720	$25,080
**Efficiency**				
**Cost per patient tested**	$1,099	$1,649	$1,184	$1,775
**Cost per hereditary cancer panel case detected**	$5,251	$7,877	$5,821	$8,729

Table S10. Scenario analysis on personnel time as a percentage of base-case personnel time.

	KPWA		KPMAS	
(Percentage of Base-Case Personnel Time)	80%	120%	80%	120%
**Costs**				
**Total Costs**	$111,717	$124,636	$82,542	$93,670
**Ovarian cancer Traceback cascade testing program variable costs**	$94,997	$99,556	$65,821	$68,590
**Ovarian cancer Traceback cascade testing program fixed costs**	$16,720	$25,080	$16,720	$25,080
**Efficiency**				
**Cost per patient tested**	$1,299	$1,449	$1,386	$1,573
**Cost per hereditary cancer panel case detected**	$6,205	$6,923	$6,815	$7,734

Table S11. Scenario analysis on costs and personnel time as a percentage of base-case costs and base-case personnel time.

	KPWA		KPMAS	
(Percentage of Base-Case Costs and Personnel Time)	80%	120%	80%	120%
**Costs**				
**Total Costs**	$89,374	$149,563	$66,040	$112,394
**Ovarian cancer Traceback cascade testing program variable costs**	$75,998	$119,467	$52,664	$82,298
**Ovarian cancer Traceback cascade testing program fixed costs**	$13,376	$30,096	$13,376	$30,096
**Efficiency**				
**Cost per patient tested**	$1,039	$1,739	$1,109	$1,888
**Cost per hereditary cancer panel case detected**	$4,964	$8,308	$5,453	$9,280
